# Danhong Injection Alleviates Mechanical Allodynia via Inhibiting ERK1/2 Activation and Elevates BDNF Level in Sciatic Nerve in Diabetic Rat

**DOI:** 10.1155/2018/5798453

**Published:** 2018-01-14

**Authors:** Qi Wang, Zhuang-Li Guo, Ge-Le Aori, Da-Wei Kong, Wen-Qiang Yang, Li Zhang, Yan-Bing Yu

**Affiliations:** ^1^Peking University China-Japan Friendship School of Clinical Medicine, Beijing, China; ^2^Department of Neurosurgery, China-Japan Friendship Hospital, Beijing, China; ^3^Department of Rehabilitation, The Affiliated Hospital of Qingdao University, Qingdao, China

## Abstract

Danhong injection (DHI) has been widely used in China for cardiocerebrovascular diseases treatments. And in this study, we demonstrated the therapeutic effect of DHI on experimental diabetic neuropathy for the first time.* Methods*. Streptozotocin- (STZ-) induced SD rats were used. In experiment 1, 4-week treatment with DHI or saline started 4 weeks after STZ injection; mechanical allodynia was measured before and every 2 weeks after STZ injection. In experiment 2, chronic intrathecal infusion of U0126 was conducted during the 8th week of diabetes. Phosphorylated and total ERK1/2 in spinal cord were analyzed by western blot. BDNF level in sciatic nerve was evaluated by ELISA.* Results*. DHI treatment significantly alleviated mechanical allodynia at the end of the study and downregulated the expression of phosphorylated ERK1/2 in spinal cord. In addition, DHI treatment also elevated brain-derived neurotrophic factor (BDNF) level in sciatic nerve of DPN rat. In experiment 2, inhibition of ERK1/2 activation was confirmed to result in the alleviation of mechanical allodynia.* Conclusions*. We demonstrated that DHI was able to alleviate mechanical allodynia in diabetic neuropathy rat through inhibiting the activation of ERK1/2. The reduction of BDNF content in sciatic nerve was also partially reversed by DHI treatment.

## 1. Introduction

Diabetic peripheral neuropathy (DPN) is a major complication of diabetes mellitus (DM) [1]. A third of patients with DPN develop painful symptoms [2]. Painful DPN dramatically impact patients' quality of life (QoL) compared with painless DPN [3]. However, the current pharmacological treatments are not entirely satisfactory and the side effects are relatively frequent [4]. The pathogenesis of diabetic neuropathic pain is still not clear. Previous studies demonstrated that mitogen-activated protein kinases (MAPKs) including extracellular signal-regulated protein kinase 1/2 (ERK1/2), p38-MAPK (p38), and c-Jun N-terminal kinase (JNK) are correlated with diabetic hyperalgesia in three-week-old diabetic, hyperalgesic rats [5]. Therefore, they may be potential target for diabetic neuropathic pain treatment.

Decreased neurotrophic support is believed to be one of the important mechanisms in the development of DPN [1]. Brain-derived neurotrophic factor (BDNF) is a member of the neurotrophin family and is critical for the survival and regeneration of neurons in peripheral nervous system. Several studies have reported that the level of BDNF in sciatic nerves decreased significantly in experimental DPN models [6, 7]. Study showed that exogenous BDNF was able to alleviate the impairment of sciatic nerve in young DPN rats [8].

Danhong injection (DHI) is a Chinese Materia Medica standardized product approved by State Food and Drug administration of China (Permission number Z20026866) [9]. The effective constituents are extracted from* Radix Salviae miltiorrhizae* and* Flos Carthami tinctorii *[10, 11]. Although it has been widely used in Chinese hospitals and clinics with proven efficacy and safety for the treatment and prophylaxis of various cardiocerebrovascular diseases [12–14], its therapeutic indications and mechanisms of effects still need to be further discovered.

Recent study has demonstrated that DHI was able to inhibit the development of diabetic retinopathy and nephropathy [15]. In the present study, we tested the effect of DHI on pain syndrome in a rat model of DPN and try to find out the underlining pathways involved in the effect. The effect of DHI on the level of BDNF in sciatic nerve was also evaluated.

## 2. Materials and Methods

### 2.1. Animal Experiments

This study was approved by the Committee on the Ethics of Animal Experiments at China-Japan Friendship Hospital. All procedures strictly complied with the Principles of Laboratory Animal Care and the Guide for the Care and Use of Laboratory Animals. Male Sprague-Dawley rats (180–200 g) were purchased from the Beijing HFK Bioscience Co. Ltd. The animals were kept two per cage under a 12-hour light/dark cycle and fed on standard laboratory diet and tap water ad libitum in a temperature- and humidity-controlled room. Rats were acclimated for 1 week before diabetes was induced by a single injection of STZ (60 mg/kg body weight) intraperitoneally after an overnight fast. Control rat received the same volume of citric acid buffer. 72 hours later, blood was taken from the tail vein for glucose concentration measurement. Rats with nonfasting blood glucose concentrations > 16.7 mmol/l were included in the following experiments.

In experiment 1, to evaluate the effect of DHI on mechanical allodynia in rats caused by diabetes, diabetic rats were randomly divided into DPN group and DHI treated group. Each group contained 9 rats. Diabetic rats were injected with saline (5 ml/kg body weight, DPN + saline group) or DHI (5 ml/kg body weight, DPN + DHI group) daily intraperitoneally. The 4-week treatment started 4 weeks after induction of diabetes. Age- and sex-matched nondiabetic-control rats received the same amount of saline. The blood glucose concentration and body weight were measured at the end of the experiment and mechanical allodynia was measured every two weeks.

In experiment 2, the role that ERK1/2 plays in the mechanical allodynia was evaluated. 18 rats with 7-week STZ-induced diabetes were used. Mitogen-activated protein kinase (MAPK) kinase 1/2 inhibitor U0126 was delivered intrathecally with a sustained rate of 0.5 *μ*g·*μ*l^−1^·hr^−1^ during the 8th week of diabetes. 10% DMSO in artificial CSF (126.6 mM NaCl, 10.0 mM NaHCO3, 2.5 mM KCl, and 2.0 mM MgCl2) was used as vehicle control. The blood glucose concentration, body weight, and mechanical allodynia were measured at the end of the experiment.

### 2.2. Behavior Study

Rats were placed on stainless steel mesh floor and were acclimatized in individual plexiglass boxes for 30 minutes. The von Frey filaments (range 1.4–26 g, North Coast Medical Inc., CA) were applied perpendicularly to the plantar surface of the hind paw and were bended with a sustained force for 6 s. The presence of brisk withdrawal or paw flinching was regarded as positive response. Every stimulus was applied with an interval of 15 seconds. A total of 3 trials were conducted on every rat with 20 minutes interval. An up and down method was used to calculate 50% paw withdraw threshold [16, 17].

### 2.3. Intrathecal Infusion

Rats were anaesthetized with sodium pentobarbital (50 mg/kg, i.p.) and immobilized with a stereotactic device. Intrathecal catheters were inserted into subarachnoid spaces through atlantooccipital membrane; the tips were carefully advanced 8 cm caudally to the position of lumbar enlargement. Infusion pumps (Model 1007D, ALZET, flow rate of 1 *μ*l·hr^−1^) filled with U0126 or vehicle were attached to the end of the catheter and fixed subcutaneously. Incisions were sealed with silk suture. Rats without any neurological deficit were included.

### 2.4. Tissue Collection

After 8 weeks of diabetes, rats were anaesthetized with sodium pentobarbital and decapitated. Dorsal parts of the lumbar enlargements of the spinal cords and sciatic nerves were collected and snap frozen in liquid nitrogen. Tissues were stored at −80°C before use.

### 2.5. Western Blot

Lumbar enlargements of the spinal cords (L4–L6) were homogenized with glass homogenizers on ice in lysis buffer (50 mM Tris-HCl, pH = 7.4, 150 mM NaCl, 1% NP-40, 5 mM EDTA) supplemented with protease and phosphatase inhibitor cocktail (Thermo Scientific) and PMSF (Beyotime). The homogenates were then centrifuged at 12000 rpm for 5 minutes at 4°C. The supernatants were collected and protein concentration was determined by BCA assay kit (Beyotime). 20 *μ*g protein was separated by 10% SDS-PAGE and transferred to polyvinylidene fluoride membrane (Thermo Scientific). The membranes were blocked with blocking buffer (Beyotime) and then incubated with rabbit monoclonal antiphosphorylated ERK1/2 antibody (dilution 1 : 2000, Cell Signaling Technology) overnight at 4°C with shaking. Horseradish peroxidase-conjugated goat anti-rabbit polyclonal antibody (dilution 1 : 1000, Beyotime) was applied at room temperature for 1 hour. The blots were visualized with enhanced chemiluminescence substrate (Thermo Scientific) in a gel imager (Bio-Rad). For total ERK1/2 detection, membranes were incubated again with rabbit monoclonal anti-ERK1/2 antibody (dilution 1 : 1000, Cell Signaling Technology) after stripping and blocking. Band density was measured with ImageJ software. Expression of phosphorylated ERK1/2 was corrected for by total ERK1/2 expression from the same sample. Quantitation of the expression of phosphorylated ERK1/2 was represented as fold change compared with control group or vehicle group.

### 2.6. Real-Time PCR

Total RNA were extracted from spinal cords (L4–L6) using TRIzol reagent (Invitrogen) according to manufacturer's protocol. FastQuant RT Kit (TIANGEN) was used for cDNA synthetization. Quantitative PCR reactions were done on Applied Biosystems 7500 with SuperReal Color PreMix reagent (TIANGEN) according to manufacturer's instructions. ΔCT was calculated using the following formula: ΔCT = CT_ERK1/2_ − CT_*β*-actin_. Results were expressed as fold changes related to control group. Primers for ERK1, ERK2, and *β*-actin used in the study were as follows:

**Table:** 

ERK1;	5′-GGACCTGAAGCCCTCCAATC-3′
	3′-GGGCAAGGCCAAAATCACAG-5′
ERK2;	5′-AATGTTCTGCACCGTGACCT-3′
	3′-TGGTCTGGATCTGCAACACG-5′
*β*-actin;	5′-TACCCCATTGAACACGGCAT-3′
	3′-GGACAACACAGCCTGGATGG-5′

### 2.7. BDNF Assay

Total protein was prepared from sciatic nerves in the way similar with that mentioned in the western blot part. BDNF level was measured by BDNF ELISA kit (*E*max Immunoassay System; Promega) according to manufacturer's instruction and was normalized to protein content that was loaded. Result was expressed as mean percentage of BDNF level found in control rats, which was denoted as 100%.

### 2.8. Statistical Analysis

All data are presented as mean ± SE. Data analysis was done with SPSS (Version 20.0; IBM Corp, Armonk, NY) software. Normality of data was assessed by 1-sample Kolmogorov-Smirnov test. Data were compared among groups with one-way ANOVA followed by post hoc Bonferroni correction in experiment 1. In experiment 2, data were compared with Student's* t*-test between the two groups. A *p* value < 0.05 was considered statistical significant.

## 3. Results

Table 1 shows the baseline and final body weights and blood glucose levels. In experiment 1, final body weights were significantly higher in control group than in DPN group (*p* < 0.01) and DHI treated group (*p* < 0.01); final blood glucose levels were around four times higher in DPN group (*p* < 0.01) and DHI treated group (*p* < 0.01) than in control group; no significant difference was found in final body weights and blood glucose levels between DPN group and DHI treated group. In experiment 2, no significant difference was found between DPN + U0126 rats and DPN + vehicle rats in baseline and final body weights and blood glucose levels.

**Figure 1:**
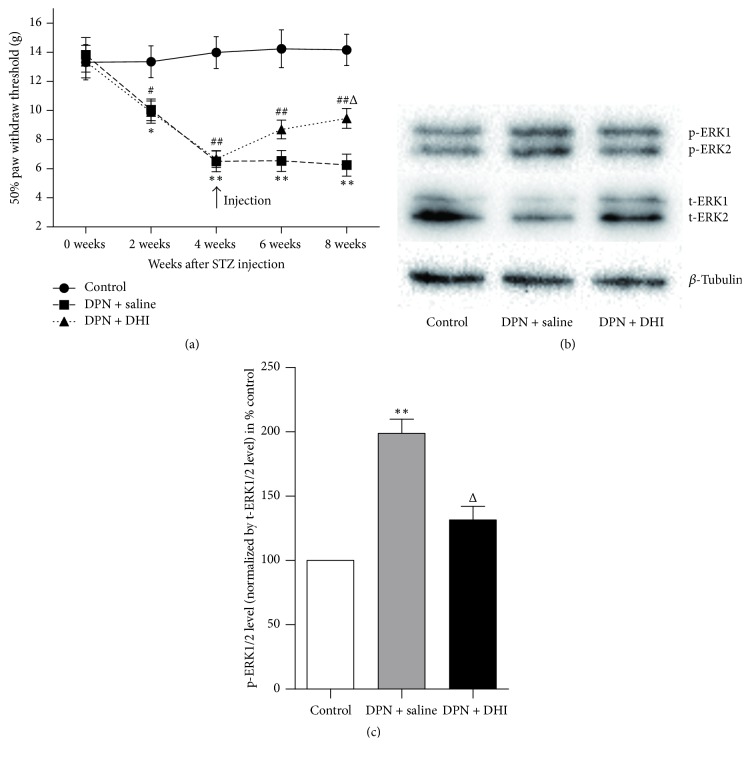
Effect of DHI on 50% paw withdraws threshold and ERK1/2 activation in spinal cord after STZ-induced diabetes. DHI or saline treatment was started after 4 weeks of diabetes. (a) Alterations in 50% paw withdraw threshold. (b) Representative western blot result of phosphorylated and total ERK1/2. (c) Phosphorylated ERK1/2 level was first corrected for by total ERK1/2 level and expressed as % control. ^*∗*^*p* < 0.05 and ^*∗∗*^*p* < 0.01 versus control group; ^#^*p* < 0.05 and ^##^*p* < 0.01 versus control group; ^Δ^*p* < 0.05 and *p* < 0.01 versus DPN + saline group. *n* = 8~9.

**Figure 2:**
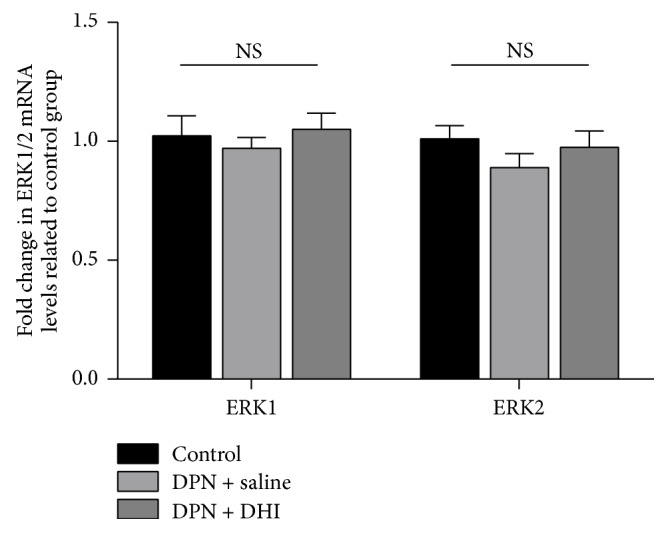
The results of ERK1/2 mRNA levels in control group, DPN + saline, and DPN + DHI group. There was no significant difference between these groups. *n* = 8~9.

The 50% withdraw thresholds reduced significantly since the 2nd week after STZ injection (*p* < 0.05). After 4 weeks of DHI treatment, reduction in 50% withdraw threshold was partially reversed (Figure 1(a)). In order to demonstrate possible pathway involved in the effect of DHI treatment on mechanical allodynia in DPN rat, level of total ERK1/2, phosphorylated ERK1/2 in spinal cord was examined. Western blot showed that level of phosphorylated ERK1/2 in spinal cord was increased in DPN rat and DHI treatment was able to reverse the increased level of phosphorylated ERK1/2 (Figures 1(b) and 1(c)). ERK1/2 mRNA levels were also evaluated. Real-time PCR demonstrated that ERK1/2 mRNA levels were not significantly different between groups (Figure 2).

**Figure 3:**
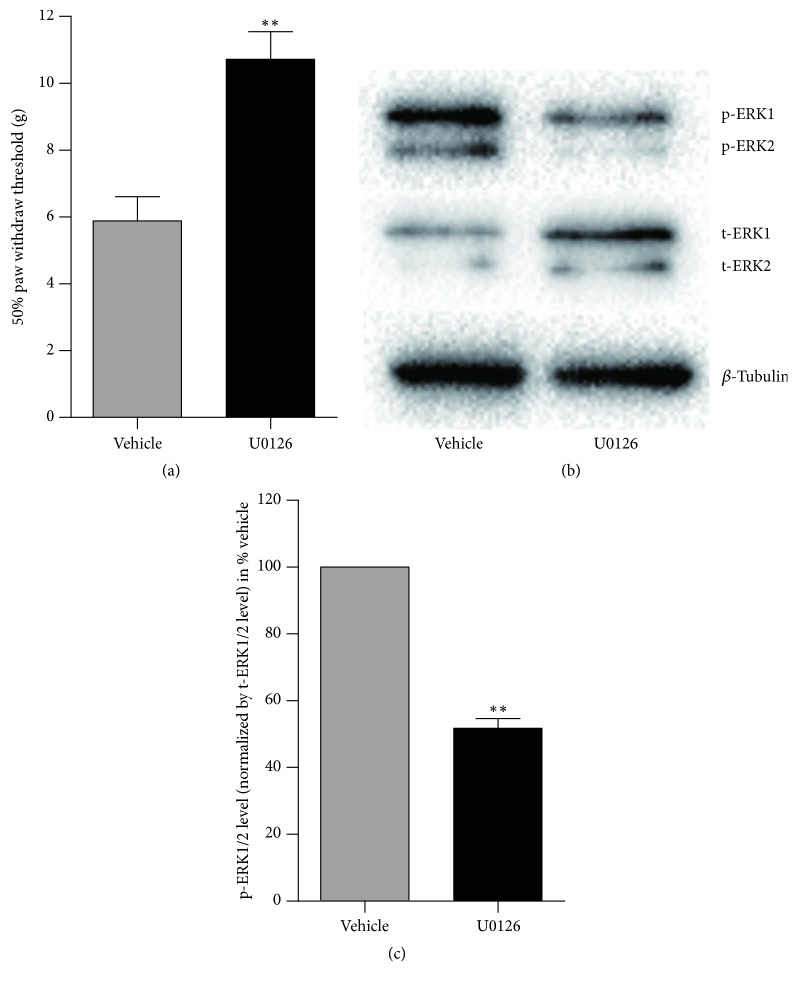
Effect of intrathecal U0126 infusion on mechanical allodynia caused by DPN and confirmation of the inhibition of ERK1/2 activation in spinal cord by intrathecal U0126 injection. (a) 50% paw withdraw thresholds in vehicle and U0126 treated groups. (b) Representative western blot result of p-ERK1/2 and t-ERK1/2. (c) Phosphorylated ERK1/2 level was first corrected for by total ERK1/2 level and expressed as % DPN + vehicle. ^*∗∗*^*p* < 0.01 versus DPN + vehicle group. *n* = 8.

To confirm that the increased phosphorylated ERK1/2 in spinal cord contributes to mechanical allodynia in DPN rat, sustained intrathecal infusion with U0126 was performed to inhibit the phosphorylation process of ERK1/2. After 1 week of U0126 infusion, the level of phosphorylated ERK1/2 in spinal cord of DPN rat was significantly reduced. The mechanical allodynia was also alleviated in DPN + U0126 group significantly compared with DPN + vehicle group (*p* < 0.01) (Figure 3).

**Figure 4:**
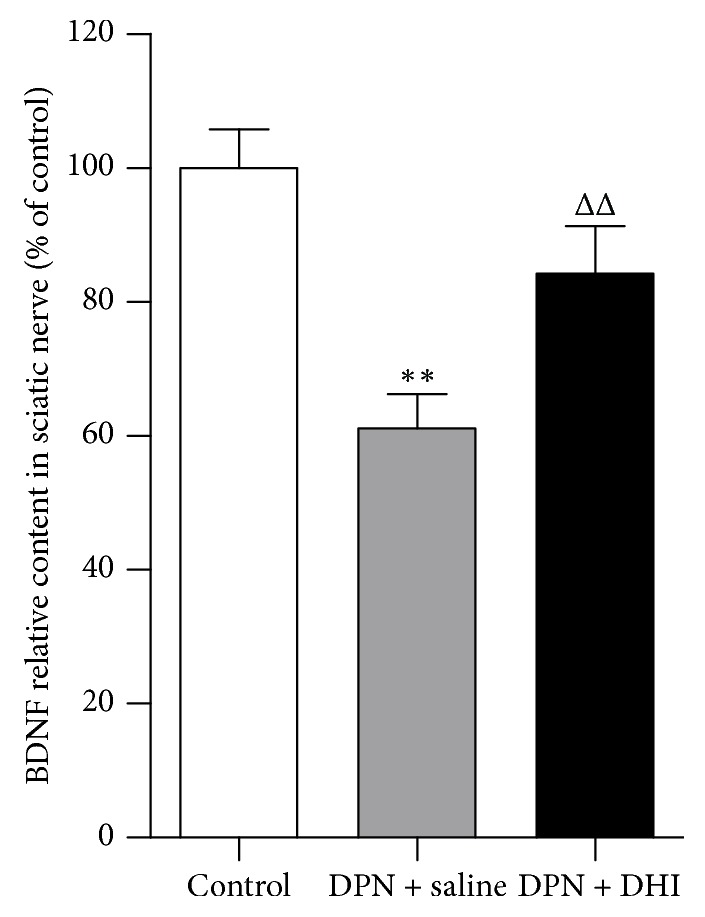
Effect of DHI on BDNF level in sciatic nerve of DPN rat. ^*∗∗*^*p* < 0.01 versus control group. ^ΔΔ^*p* < 0.01 versus DPN + saline group. *n* = 8~9.

The BDNF level in sciatic nerve in each group was expressed as fold change related to control group. BDNF decreased significantly in DPN group compared with control group (*p* < 0.01), and DHI treatment elevated BDNF level significantly compared with saline (*p* < 0.01) (Figure 4). Additionally, there was no significant difference between U0126 treated group and DPN + vehicle group in experiment 2 (data not shown).

## 4. Discussions

DHI was initially developed to treat various cardiocerebrovascular diseases according to traditional Chinese medical theory [18–20]. In present study, we proved that DHI alleviated mechanical allodynia through inhibiting the activation of ERK1/2 pathway in spinal cord and elevated the level of BDNF in sciatic nerves in DPN rats.

Mechanical allodynia is a common neuropathic pain syndrome of DPN patients. In this STZ-induced DPN rat model, mechanical allodynia was observed 2 weeks after STZ injection as previously described [21]. ERK1/2 is a member of mitogen-activated protein kinase (MAPK) family and is activated by phosphorylation. Activation of ERK1/2 in spinal cord was thought to play a role in the generation of neuropathic pain in DPN rats [21, 22]. Meanwhile some others argued that diabetes-induced activation of ERK1/2 is a protective reaction of stressed nerve cells [23, 24]. In this study, we found 2 weeks of DHI treatment ameliorated the mechanical allodynia and the effect was accompanied by reduced activation of ERK 1/2 in spinal cord. We further confirmed that reduced activation of ERK1/2 resulted in the alleviation of mechanical allodynia by continuous intrathecal injection of specific ERK1/2 inhibitor. Therefore, treatment effect of DHI on mechanical allodynia could be or, at least in part, attributed to the inhibitory property on ERK1/2 activation.

Loss of neurotrophic support is believed to involve in the pathogenesis of DPN. Several clinical trials have tried to supply DPN patients with recombinant human NGF or BDNF to study their effects on neuropathic deficits caused by DPN. But they both failed to achieve positive effects [25, 26]. It is believed that systemic administration of NGF or BDNF might not achieve its local, sufficient, and sustained effects [7, 27]. Additionally high rate of the presence of hyperalgesia at injection site is also a troublesome issue [25]. Thus, medications which could raise the level of endogenous neurotrophic factors may be ideal therapies [7, 27]. In the present study, we proved that DHI increased BDNF level in sciatic nerve of DPN rats. Further studies may be conducted to evaluate the role of DHI as an endogenous BDNF stimulator.

To our knowledge, previous researches on mechanisms involved in the reduction of neurotrophic factors were almost all in vitro studies. In most of these studies, Schwann cells were exposed to high glucose culture medium to achieve an experimental DPN model. However, heterogeneous results, decreased, unchanged, and increased expression of neurotrophic factors by Schwann cells exposed to high glucose condition, were reported [28–31]. Therefore, high glucose treated Schwann cells may not be an appropriate model mimicking the real disease conditions in vivo. Future in vivo studies may be preferred to elucidate the mechanisms of DPN-induced reduction of neurotrophic factors in sciatic nerve. Sustained delivery of various inhibitors through intrathecal catheters may be a suitable method to find out possible pathways involved. In the present study, intrathecal injection of ERK1/2 inhibitor was found unable to influence the level of BDNF in sciatic nerves of DPN rats. Other inhibitors may be examined.

## 5. Conclusions

ERK1/2 activation in spinal cord contributes to mechanical allodynia in STZ-induced DPN rat. DHI alleviates the mechanical allodynia by the inhibition of ERK1/2 activation. In addition, DHI treatment reverses the reduction of BDNF in sciatic nerve in DPN rat. This study extends the potential indication for DHI which has been conventionally used for cardiocerebrovascular diseases.

## Figures and Tables

**Table 1:** Baseline and final body weights and final blood glucose levels in different groups.

	Body weight (g)		Blood glucose (mmol/l)	
	Baseline	Final	Baseline	Final
Experiment 1				
Control	256.25 ± 2.53	523.63 ± 5.64	6.68 ± 0.13	6.73 ± 0.16
DPN + saline	257.22 ± 1.71	334.11 ± 7.57^*∗∗*^	6.72 ± 0.09	31.12 ± 1.03^*∗∗*^
DPN + DHI	258.11 ± 2.02	339.90 ± 6.52^*∗∗*^	6.72 ± 0.14	30.06 ± 1.00^*∗∗*^
Experiment 2				
DPN + vehicle	258.25 ± 2.26	329.75 ± 5.81	6.60 ± 0.13	29.75 ± 0.84
DPN + U0126	257.75 ± 1.75	320.63 ± 6.32	6.48 ± 0.16	29.94 ± 0.89

^*∗∗*^ *p* < 0.01 versus control.
